# Estimating the frequency and characteristics of respiratory disease outbreaks at mass gatherings in the United States: Findings from a state and local health department assessment

**DOI:** 10.1371/journal.pone.0186730

**Published:** 2017-10-27

**Authors:** Argelia Figueroa, Reena K. Gulati, Jeanette J. Rainey

**Affiliations:** 1 Division of Global Migration and Quarantine, Centers for Disease Control and Prevention, Atlanta, Georgia, United States of America; 2 Division of Global Health Protection, Centers for Disease Control and Prevention, Atlanta, Georgia, United States of America; University of Washington, UNITED STATES

## Abstract

Mass gatherings create environments conducive to the transmission of infectious diseases. Thousands of mass gatherings are held annually in the United States; however, information on the frequency and characteristics of respiratory disease outbreaks and on the use of nonpharmaceutical interventions at these gatherings is scarce. We administered an online assessment to the 50 state health departments and 31 large local health departments in the United States to gather information about mass gathering-related respiratory disease outbreaks occurring between 2009 and 2014. The assessment also captured information on the use of nonpharmaceutical interventions to slow disease transmission in these settings. We downloaded respondent data into a SAS dataset for descriptive analyses. We received responses from 43 (53%) of the 81 health jurisdictions. Among these, 8 reported 18 mass gathering outbreaks. More than half (n = 11) of the outbreaks involved zoonotic transmission of influenza A (H3N2v) at county and state fairs. Other outbreaks occurred at camps (influenza A (H1N1)pdm09 [n = 2] and A (H3) [n = 1]), religious gatherings (influenza A (H1N1)pdm09 [n = 1] and unspecified respiratory virus [n = 1]), at a conference (influenza A (H1N1)pdm09), and a sporting event (influenza A). Outbreaks ranged from 5 to 150 reported cases. Of the 43 respondents, 9 jurisdictions used nonpharmaceutical interventions to slow or prevent disease transmission. Although respiratory disease outbreaks with a large number of cases occur at many types of mass gatherings, our assessment suggests that such outbreaks may be uncommon, even during the 2009 influenza A (H1N1) pandemic, which partially explains the reported, but limited, use of nonpharmaceutical interventions. More research on the characteristics of mass gatherings with respiratory disease outbreaks and effectiveness of nonpharmaceutical interventions would likely be beneficial for decision makers at state and local health departments when responding to future outbreaks and pandemics.

## Introduction

Mass gatherings may create conducive settings for infectious disease transmission, including pandemic influenza [1–7]. Influenza is most commonly transmitted through infectious respiratory droplets and contact with secretions or fomites [8], which typically requires close contact between susceptible and infectious persons. Thousands of mass gatherings are held annually in the United States (for example, more than 14,000 day and resident camps [9] and nearly 1.8 million meetings [10]). Since mass gatherings bring thousands of persons together in a finite geographic area for a shared event or experience [3–5], these events could lead to rapid transmission and propagation of respiratory infections, including pandemic influenza.

During a pandemic, public health officials must prioritize activities that slow or mitigate the transmission of the infectious agent [5]. The U.S. Centers for Disease Control and Prevention (CDC) recently revised pre-pandemic planning guidelines for preparing, planning for, and responding to a pandemic of influenza or other novel respiratory disease for a variety of settings, including mass gatherings [11]. CDC routinely recommends that state and local health departments implement a variety of nonpharmaceutical interventions (NPIs), including hand hygiene, respiratory etiquette, and environmental surface cleaning, to reduce disease transmission in mass gatherings. During a severe pandemic, CDC may recommend social distancing and postponing or canceling mass gatherings [11]. Additional information on the characteristics of mass gatherings posing the greatest risk for acute respiratory outbreaks could help strengthen this guidance and better inform state and local public health decision makers when preparing for future pandemics.

Findings from a recently conducted systematic literature review suggest that mass gathering-related respiratory disease outbreaks are rare in the United States [12]. However, outbreaks at agricultural fairs (i.e., exposure to infected swine) and at camps where participants had close social contact in communal housing have been reported [12–16]. Participant density and susceptibility, rather than gathering size alone, could be relevant factors in predicting the occurrence of outbreaks in these settings [2, 3, 5].

We conducted an assessment among state and local public health officials to obtain additional information on mass gathering-related respiratory disease outbreaks occurring in the United States between January 1, 2009 and December 31, 2014. The assessment’s objectives included: 1) describing the frequency and characteristics of mass gathering-related respiratory disease outbreaks reported by state and local health departments and 2) identifying the types of strategies or approaches used for outbreak prevention and control in mass gatherings. We implemented the project in collaboration with the Council of State and Territorial Epidemiologists (CSTE) and the National Association of County and City Health Officials (NACCHO).

## Methods

### Target population

We developed an online assessment on mass gathering-related respiratory disease outbreaks and the prevention and control strategies implemented at these settings and invited public health officials at 50 state health departments and 31 large local health departments (each serving populations > 250,000) in the country to respond. Given their population size, these jurisdictions were most likely to have mass gatherings (e.g., sporting events, conferences, music festivals, fairs) that met our inclusion criteria as well as the staff necessary to investigate and report on possible outbreaks.

### Data collection and analysis

The online assessment consisted of two parts (S1 Supplement). The first part requested information on respiratory disease outbreaks of related to mass gatherings occurring in the United States between January 1, 2009 and December 31, 2014, including the size and etiology of the outbreak, and the characteristics of the mass gathering (purpose, dates, venue type, and size). An outbreak was defined as one or more cases of an infectious respiratory disease associated with a mass gathering (transmission linked to a point source, animal, or another person at the mass gathering). Although a mass gathering was defined as a planned or unplanned congregation of 1,000 or more persons in either an indoor or an outdoor venue for a common purpose, we included all outbreaks reported in response to the assessment regardless of the gathering’s size. The second part of the assessment requested information on the use of NPIs, public health messaging, and on-site surveillance by health departments for mass gathering events in general. We also asked for respondent recommendations on improving CDC support for state and local health departments.

Invitation emails with a link to the online assessment were sent to officials at the state health departments in March 2016 and to officials at the local health departments in April 2016. Respondents had 4 weeks to complete the assessment and received two reminder emails during that time. Responses were automatically saved when entered, and respondents could pause the assessment and revise their entries within the 4-week assessment period. We followed up with respondents to verify the reported outbreaks and obtained supplemental event information from publicly available websites. All responses (partially and fully completed) were saved on a CDC server. At the end of the 4-week assessment period, data were downloaded and saved as a SAS^®^ (version 9.3) database for analysis.

We combined the assessment data from fully completed and partially completed assessments and deleted any identified duplicate outbreak reports. Descriptive analyses were conducted to estimate: 1) the number of mass gathering-related respiratory disease outbreaks reported from 2009 to 2014, 2) the etiologies of the outbreaks, and 3) the characteristics of the mass gatherings. For this project, we presumed that all mass gathering participants were susceptible to influenza regardless of previous vaccination or infection. We estimated attack rates for viruses involving person-to-person transmission by dividing the number of confirmed and probable cases by the approximate size of the gathering. Respiratory infections involving zoonotic transmission require direct or indirect exposure to the animal host. Since we were unable to approximate the number of fair attendees visiting the swine exhibits, we elected not to estimate the attack rates for reported influenza H3N2v outbreaks. We also described surveillance, intervention, and communication strategies that respondents used for preventing or controlling infectious disease transmission in mass gatherings. We qualitatively grouped respondents’ recommendations for improving current CDC support into common themes.

### Ethical review and approval

The project protocol and data collection tools were reviewed and approved by CSTE’s Infectious Disease Committee and by NACCHO. An advisor from CDC’s Human Research Protection Office reviewed and approved the project as non-research. The Office of Management and Budget reviewed and approved the project under the Paperwork Reduction Act of 1995 for information collection requests by federal agencies (OMB #0920–0879). Participation was voluntary. Consent was implied if a respondent voluntarily answered the online assessment. The assessment only gathered information about the outbreak and the mass gathering in aggregate form; personally identifiable information about case-patients was not obtained.

## Results

We received 31 (38%) fully completed and 12 (15%) partially completed assessments from the 50 state health departments and 31 local health departments, resulting in 43 (53%) responses received during the project period (Table 1). Eight of the 27 state health departments submitting fully completed assessments reported a total of 18 mass gathering-related respiratory disease outbreaks. None of the local health departments reported outbreaks, and no duplicate outbreaks were identified. Information on the use of NPIs and other preventive approaches was obtained from 32 respondents.

**Table 1:** Number of state and local health departments responding to an online assessment of mass gathering-related respiratory disease outbreaks, United States, 2009–2014. Assessment administered in March–April 2016.

	Total (%)^a^ (N = 81)	State (%) (N = 50)	Local (%) (N = 31)
**Fully completed**	31 (38)	27 (54)	4 (13)
**Partially completed**^b^	12 (15)	7 (14)	5 (16)
**Total**	43 (53)	34 (68)	9 (29)

^a^Assessment administered to all 50 state health departments and 31 of the largest local health departments in the United States (includes city and county health offices).

^b^Responses were automatically entered and saved on a Centers for Disease Control and Prevention server; respondents could revise and/or add information any time during the assessment period.

### Mass gathering outbreaks

Of the 18 reported mass gathering-related outbreaks, 3 occurred in 2009, one in 2010, 2 in 2011, 11 in 2012, and 1 in 2013. All outbreaks involved multi-day gatherings. Most (94%) involved influenza A and occurred at fairs, camps, religious gatherings, a conference-related social gathering, and a sporting event (Table 2). These mass gatherings took place at indoor or indoor-outdoor venues. Outbreaks ranged in size from 5 to 150 reported cases.

**Table 2:** Characteristics of mass gathering-related respiratory disease outbreaks, United States, 2009–2014. Reported by eight state health departments through an online assessment, March—April 2016.

Outbreak Number^§^	Year	Pathogen	Transmission	Type of Gathering	Annual Event (Y/N)	Season^a^	Duration of Gathering (Days)	Number of Attendees (Size of Gathering)	Number of Cases^b^	Age of Cases (Years)	Attack Rate (%)^c^
1	2009	Influenza A (H1N1) pdm09	Person-to- person	Camp	Y	Summer	14	2,850	40	Unknown	1.4
2	2009	Influenza A (H1N1) pdm09	Person-to-person	Camp	Y	Summer	7	1,300	62	2–64	4.8
3	2009	Influenza A (H1N1) pdm09	Person-to- person	Religious event	N	Summer	8	630	123	2–64	19.5
4	2010	Respiratory virus unspecified	Person-to- person	Religious event	N	Summer	Unknown	3,700	5	18–64	0.1
5	2011	Influenza A (H1N1) pdm09	Person-to- person	Professionalconference^d^	N	Winter	3	715	150	18–64	21.0
6	2011	Influenza A	Person-to- person	Sporting event	Y	Spring	4	3,900	130	Unknown	3.3
7	2012	Influenza A (H3N2v)	Animal-to- person (zoonotic)	Fair	Y	Summer	30	300,000	138	Infants through 65+	Unknown
8	2012	Influenza A (H3N2v)	Animal-to- person (zoonotic)	Fair	Y	Summer	7	95,000	78	Unknown	Unknown
9	2012	Influenza A (H3N2v)	Animal-to- person (zoonotic)	Fair	Y	Summer	7	30,000	28	Unknown	Unknown
10	2012	Influenza A (H3N2v)	Animal-to- person (zoonotic)	Fair	Y	Summer	7	31,000	25	Unknown	Unknown
11	2012	Influenza A (H3N2v)	Animal-to- person (zoonotic)	Fair	Y	Summer	7	60,000	22	Unknown	Unknown
12	2012	Influenza A (H3N2v)	Animal-to- person (zoonotic)	Fair	Y	Summer	7	85,000	15	Unknown	Unknown
13	2012	Influenza A (H3N2v)	Animal-to- person (zoonotic)	Fair	Y	Summer	7	80,000	12	Unknown	Unknown
14	2012	Influenza A (H3N2v)	Animal-to- person (zoonotic)	Fair	Y	Summer	7	160,000	10	Unknown	Unknown
15	2012	Influenza A (H3N2v)	Animal-to- person (zoonotic)	Fair	Y	Summer	7	80,000	6	Unknown	Unknown
16	2012	Influenza A (H3N2v)	Animal-to- person (zoonotic)	Fair	Y	Summer	7	40,000	4	Unknown	Unknown
17	2012	Influenza A (H3N2v)	Animal-to- person (zoonotic)	Fair	Y	Summer	7	87,000	3	Unknown	Unknown
18	2013	Influenza A (H3)	Person-to-person	Camp	N	Summer	10	52,319	10	2–17	0.02

^§^Outbreaks 1 and 2 were reported by one state. Outbreaks 8 to 17 were reported by another state. The latter state experienced two additional fair-related influenza A (H3N2v) outbreaks in 2012, but the assessment tool limited the number of outbreaks reported to 10. Outbreaks 3 and 5 were included because of their high attack rates. Outbreaks 1 to 3 occurred during the 2009 influenza A (H1N1) pandemic.

^a^Based on the month of occurrence of reported outbreak.

^b^Represents the total number of confirmed and probable cases.

^c^For viruses with person-to-person transmission, the attack rate was estimated as the number of reported cases divided by the approximate size of the mass gathering (number of attendees), assuming that all participants were susceptible at the beginning of the gathering. Since we were unable to approximate the number of fair attendees with probable swine contact or who were in close proximity to the swine exhibit, we elected not to estimate attack rates for the fair-related influenza A (H3N2v) outbreaks.

^d^More than 50% of ill conference attendees participated in social gathering held at a separate venue on the last day of a 3-day professional conference. The estimated attack rate assumes that the 715 conference attendees were equally at-risk of exposure and infection.

#### Fairs

More than half (n = 11, 61%) of the reported mass gathering-related outbreaks occurred at state or county agricultural fairs in 2012 (Table 3). These were large annual events with a duration ranging between 7 and 30 days with an average attendance of 95,200 persons. All fair-related outbreaks were reported from two neighboring states and involved zoonotic transmission of influenza A (H3N2v) following presumed contact with swine or close proximity to swine exhibits. The number of cases reported from these outbreaks ranged from 3 to 138 and occurred across all age groups.

**Table 3:** Mass gathering-related respiratory disease outbreaks by type of gathering, pathogen, and mode of transmission, United States, 2009–2014. Reported by eight state health departments through an online assessment, March 2016.

Type of Gathering	Pathogen	Mode of Transmission	Number of Outbreaks
Fairs	influenza A (H3N2v)	Zoonotic	11
Camps	influenza A (H1N1)pdm09	Person-to-person	2
	influenza A (H3)	Person-to-person	1
Religious gatherings	influenza A (H1N1)pdm09	Person-to-person	1
	unspecified respiratory virus	Person-to-person	1
Professional conference	influenza A (H1N1)pdm09	Person-to-person	1
Sporting event	influenza A (unspecified)	Person-to-person	1
**Total**			**18**

#### Camps

Three (17%) of the 18 reported mass gathering-related outbreaks occurred at recreational and scouting summer camps. These camps lasted between 7 and 14 days and had between 1,300 and 52,000 participants. Two of these outbreaks occurred at recreational camps in June and July 2009 and involved transmission of influenza A (H1N1)pdm09 with an estimated attack rate of 1.4% and 4.8%, respectively. The third outbreak occurred at a scouting camp in July 2013, involved transmission of influenza A (H3), and had an estimated attack rate of 0.02%.

#### Religious gatherings

Two (11%) of the reported mass gathering-related outbreaks occurred at religious gatherings. One of these outbreaks occurred in July 2009 at a religious gathering combining recreational and educational activities and involved transmission of influenza A (H1N1)pdm09. During the 8-day gathering, 123 of the 630 participants—all aged between 2 and 17 years—reported symptoms, resulting in an attack rate of 20%. The second outbreak took place at a religious educational gathering in September 2010 and involved transmission of an unspecified respiratory virus. During this multi-day gathering, 5 of the approximately 3,700 participants, all aged between 18 and 64 years, were reported as cases for an attack rate of 0.1%.

#### Other gatherings

In 2011, 2 outbreaks occurred at other types of mass gatherings. The first outbreak was associated with a 3-day social media and internet conference that attracted 715 attendees. Many of the ill attendees participated in a social gathering held at a separate venue on the last day of the conference. In total, 150 conference attendees were reported with either confirmed or probable influenza A (H1N1)pdm09 cases resulting in an attack rate of 21%. The second outbreak occurred at a national 4-day dog agility competition with approximately 3,900 attendees. The outbreak was associated with influenza A (subtype unspecified) transmission resulting in 130 probable cases and an attack rate of 3.3%. The majority of cases (n = 101, 78%) occurred among the 1,100 handlers of dogs competing in the event; the remaining 29 cases occurred in other attendees. Event participants stayed at various lodging facilities including neighborhood hotels and recreational vehicle sites.

### Interventions at mass gatherings

Of the 43 state and local health department respondents, 9 (21%) reported using at least one NPI at mass gatherings held during the assessment period regardless of known circulation of influenza or other respiratory infectious disease (Table 4). The most commonly reported NPI was increased availability of hand washing stations and distribution of hand sanitizer, particularly at fairs, to prevent transmission of influenza A (H3N2v). Two health departments implemented social distancing measures (in combination with other NPIs) in mass gatherings in 2009, presumably because of influenza A (H1N1)pdm09. One health department reported pre-emptively canceling a mass gathering in 2009 as a result of local transmission of influenza A (H1N1)pdm09 even though no previous mass gathering-related outbreaks had occurred in the jurisdiction. Additionally, 12 public health departments conducted on-site infectious disease surveillance during mass gatherings—most frequently at sporting and political events.

**Table 4:** Number of state and local health departments reporting implementing nonpharmaceutical interventions (NPIs) and public health messaging at mass gatherings by type of gathering, United States, 2009–2014. Reported by state and local health departments through an online assessment, March—April 2016.

Intervention	Type of Gathering	Number of Reporting Health Departments
Hand washing stations and/or distribution of hand sanitizer (NPI)^a^	Fair	4
	Festival	1
	Camp	1
	Religious gathering	1
Social distancing (NPI)^a^	Festival	1
	Sporting event	1
Public health messaging^b^	Fair	3
	Religious gathering	1
	Festival	1
	Political event	1
	Sporting event	1
	University commencement exercises	1

^a^Nine health departments reported implementing hand washing stations and/or distribution of hand sanitizer and social distancing (NPIs).

^b^Eight health departments used public health messaging to educate how to prevent disease transmission and raise community awareness.

Eight (19%) respondents indicated using public health messaging at mass gatherings. Messaging occurred most frequently at fairs in 2012 and 2014 to prevent the spread of influenza A (H3N2v) and other respiratory and enteric diseases. Messaging was also implemented at university commencements, religious gatherings, political events and a festival to prevent or mitigate transmission of infectious agents. Health department staff typically used a combination of posters, fact sheets, TV/radio messaging, and educational materials. The use of web links or widgets as social media tools for increasing public awareness was commonly reported.

Fourteen respondents provided recommendations for how CDC could help state and local health departments improve pandemic preparedness, communication approaches, and use of NPIs at mass gatherings. Four common themes were identified: 1) additional funding opportunities, 2) improved data sharing between jurisdictions, 3) additional training and technical assistance, and 4) strengthened collaborations with other organizations such as CSTE and NACCHO (Table 5).

**Table 5:** Health departments’ recommendations to the Centers for Disease Control and Prevention to help improve pandemic preparedness, communication approaches, and use of nonpharmaceutical interventions at mass gatherings, using four common themes. Provided by 14 state and local health departments through an online assessment, March—April 2016.

Theme	Recommendations	Number of Health Departments Making Recommendations
**Funding opportunities**	Additional funding to support program and emergency preparedness efforts Include mass gathering-related outbreaks as required topic in preparedness grant applications Provide greater flexibility in use of funds received through federal grants and cooperative agreements (e.g., staff support)	3
**Data sharing agreements**	Ensure collaboration and communication with other agencies in order to gain access to data used in outbreak investigations Provide information about outbreaks occurring in neighboring jurisdictions	2
**Training and technical assistance**	Additional technical assistance to support program and emergency preparedness efforts Provide guidance and communication templates Develop a better definition of mass gatherings for outbreaks Provide standardized lists or tools for monitoring and tracking confirmed cases Provide a checklist of things to consider when planning and preparing for mass gatherings, such as appropriate surveillance activities, public health messaging, or implementation of non-pharmaceutical interventions Offer workshops or educational sessions at professional conferences about investigations on mass gatherings, what other states have done, and integration of syndromic surveillance from mass gatherings	8
**Collaborations**	Collaborate closely with the Council of State and Territorial Epidemiologists (CSTE) and the National Association of County and City Health Officials (NACCHO) when creating or revising guidance documents Maintain existing CDC resources such as the Unexplained Respiratory Disease Outbreak (URDO) working group that assists U.S. and international public health partners in investigating unexplained respiratory outbreaks Facilitate more timely receipt of information from the Department of Homeland Security's National Bio-surveillance Integration Center for better notification of events before they have started	3

## Discussion

Findings from this assessment provided additional information on the occurrence and characteristics of mass gathering-related respiratory disease outbreaks in the United States. The data complement our systematic literature review on the topic [12]. In this assessment, 8 state health departments reported 18 mass gathering-related outbreaks among the thousands of gatherings that took place in the United States during the 6-year assessment period. The majority of reported outbreaks involved influenza A (H3N2v) transmission at county or state fairs as previously described [12–13], suggesting that mass gathering-related respiratory disease outbreaks, while uncommon, can still cause substantial morbidity among participants when they do occur.

Our assessment captured information about seven mass gathering-related outbreaks not found elsewhere in the literature including four involving transmission of influenza A (H1N1)pdm09. Two of the four previously unpublished pandemic-related outbreaks occurred at summer camps in 2009. As highlighted in our literature review, camp outbreaks typically involve close contact and social mixing among school-aged children in communal housing and during camp activities [12, 14–15].

The third previously unpublished mass gathering-related influenza A (H1N1)pdm09 outbreak occurred at a religious gathering in summer 2009 and resulted in 123 reported cases (attack rate of almost 20%). Religious gatherings often bring people together in social settings and involve repeated close contact through greeting and worship practices, as exemplified by an influenza outbreak during the 2008 World Youth Day in Sydney, Australia [16]. Additionally, religious gatherings can involve shared lodging in dormitory-style housing, resulting in mixing patterns consistent with previously described camp-related outbreaks [14]. The CDC’s guidance for preventing infections during the 2009 influenza A (H1N1) pandemic included recommendations on school dismissal as part of a comprehensive community mitigation effort for reducing disease transmission [17]. Some mass gatherings such as this multi-day religious gathering may have had influenza attack rates as high as, or higher than, school-associated outbreaks [18].

The fourth influenza A (H1N1)pdm09 outbreak identified in this assessment occurred at a professional conference in winter 2011. Although the 2009 pandemic was declared over in August 2010 [19], influenza A (H1N1)pdm09 remained the predominant strain circulating in the United States in 2011 [20]. The 21% attack rate from this outbreak was high given the event’s short duration, although higher attack rates have been reported at professional conferences, including the 36% clinical attack rate at the Eighth International Congress on Tropical Medicine and Malaria in Tehran, Iran, during the 1968 influenza A (H3N2) pandemic [21]. Because more than half of the reported ill attendees participated in the conference’s social gathering, the mixing patterns and transmission dynamics were likely more similar to influenza outbreaks observed at party environments such as music festivals held in Europe during 2009 [22–23] than typically observed at professional meetings or workshops. No other professional conference-related respiratory disease outbreak was identified from this assessment or from our systematic literature review [12].

In April 2011, an influenza A (unspecified subtype) outbreak occurred at a sporting event involving persons traveling from multiple locations with an estimated attack rate of 3.3%. Influenza is a frequent cause of respiratory infections among travelers. The infection might have been introduced from elsewhere, highlighting the risk of travel in the spread of infectious diseases [1, 4, 6, 24–25]. Similar to previously reported respiratory disease outbreaks associated with sporting events, disease transmission in this outbreak primarily occurred among event athletes; spectators and host communities experienced only nominal increases above baseline estimates [5, 24–25]. Clustering of cases among athletes could reflect frequent contact between infectious and susceptible persons during competition and shared lodging arrangements. It might also reflect differences in disease detection (i.e., more information is available on the health status of event athletes).

In 2013, a reported influenza A (H3) outbreak included 10 case-patients among the approximately 52,000 participants at a large summer camp held indoors. Findings from our systematic literature review suggested that influenza outbreaks in the summer months are rare, except during a pandemic [12, 26–27]. Influenza A (H3N2) was the predominant strain circulating in the United States during the 2012–2013 season [28–29].

Most of the state and local health departments responding to our assessment reported an increased number of hand washing stations and, in some cases, distribution of hand sanitizer, particularly at fairs, to prevent zoonotic transmission of influenza A (H3N2v). These measures can be implemented easily at most mass gatherings and are helpful for minimizing influenza transmission [30–31]. Only 2 respondents indicated having used a combination of social distancing measures and increased hand hygiene at mass gatherings in 2009 to slow or prevent transmission of influenza A (H1N1)pdm09. Social distancing measures such as seating restrictions or crowd control [3–5] can be challenging to implement—particularly if the measures have economic implications. Successful implementation will likely rely on well-developed communication plans [32] using a variety of media.

Health departments conducted on-site disease surveillance primarily at sporting and political events where the number of attendees was high and could have posed a considerable burden on the public health system.

Consistent with findings from our systematic literature review, none of the outbreaks reported in this assessment involved single-day mass gatherings, although a majority of mass gatherings (e.g., sporting events, music concerts) attracting more than 100,000 participants in the United States are single-day events lasting a few hours [33]. The lack of reporting may reflect limitations of common surveillance approaches to identify these outbreaks (i.e., linking spatially distinct disease reports back to single-day events, decreasing the likelihood of being detected and reported to a local health jurisdiction) [12]. Additionally, while multi-day mass gatherings may facilitate disease transmission, these types of gatherings may be helpful in identifying cases when they occur by monitoring participant attendance. On the other hand, shorter-duration gatherings could have a lower probability of disease transmission [5], particularly when held outdoors.

Our assessment had several limitations. First, the response rate was lower than expected. This lower rate could suggest competing priorities (e.g., Zika response) for state and local health departments, perceived burden in completing the assessment, not having detected or investigated outbreaks associated with mass gatherings, or declining to participate in the assessment. Therefore, responses are unlikely to be representative of all state and local health departments. The majority of state health department respondents reported only one mass gathering-related outbreak during the 6-year assessment period, while none of the 31 local health departments reported outbreaks. The percentages presented here are limited to the number of reported events and do not reflect percentages among all the mass gathering events that may have taken place during the assessment period. Second, health department staff in jurisdictions experiencing a mass gathering-related respiratory disease outbreak may have been more willing to participate in the assessment than those who did not, resulting in a possible reporting bias. Third, to minimize respondent burden, the assessment allowed for a maximum of 10 outbreaks to be reported and captured specific information for each reported outbreak. Because of that limit, information from two of the 12 outbreaks experienced by one health department was not obtained. Therefore, potentially important information describing the outbreak and the mass gathering setting (i.e., detailed descriptions of the venue, lodging, and associated participant mixing patterns), index cases, and subsequent transmission patterns were missed. Finally, reports for which the viral strain was unidentified were included based on the health department’s determination that an outbreak had occurred, even when those outbreaks could have been caused by more than one pathogen. Despite these limitations, our results, combined with those from our systematic literature review, offer important insights into the occurrence and characteristics of mass gathering-related respiratory disease outbreaks in the United States.

## Conclusions

This assessment suggests that respiratory disease outbreaks in mass gatherings are rare even during the 2009 influenza A (H1N1) pandemic. Outbreaks appear to occur at multi-day events and often involve shared lodging arrangements or communal settings. The low number of mass gathering-related respiratory disease outbreaks may partially explain the limited reported use of NPIs. Although state and local health departments have prevention and emergency preparedness programs in place, public health officials recommend CDC provide additional evidence-based guidance on the use of all NPIs at mass gatherings [34], strategies for on-site surveillance, and data sharing between states and other health organizations. Strengthening information sharing about mass gathering-related respiratory disease outbreak investigations can help public health officials identify common characteristics of these outbreaks and—combined with ongoing modeling work done at CDC on the use of NPIs—better predict when and where similar outbreaks might occur in the future. Additional studies describing the occurrence of outbreaks on single-day gatherings are needed.

## Disclaimer

Findings and conclusions in this document are those of the authors alone and do not necessarily represent the opinion or views of the Centers for Disease Control and Prevention.

## Supporting information

S1 Supplement Online assessment tool. Copy of questionnaire used in online assessment.
(DOCX)

## References

[1] Abubakar I, Gautret P, Brunette GW, Blumberg L, Johnson D, Poumerol G, et al Global perspectives for prevention of infectious diseases associated with mass gatherings. Lancet Infect Dis. 2012;12:66–74. doi: 10.1016/S1473-3099(11)70246-8 22192131 10.1016/S1473-3099(11)70246-8

[2] Chowell G, Nishiura H, Viboud C. Modeling rapidly disseminating infectious disease during mass gatherings. BMC Medicine. 2012;10:159 doi: 10.1186/1741-7015-10-159 23217051 10.1186/1741-7015-10-159 PMC3532170

[3] Johansson A, Batty M, Hayashi K, Al Bar O, Marcozzi D, Memish ZA. Crowd and environmental management during mass gatherings. Lancet Infect Dis. 2012;12:150–156. doi: 10.1016/S1473-3099(11)70287-0 22252150 10.1016/S1473-3099(11)70287-0

[4] Rashid H, Haworth E, Shafi S, Memish ZA, Booy R. Pandemic influenza: mass gatherings and mass infection. Lancet Infect Dis. 2008; 8:526–527. doi: 10.1016/S1473-3099(08)70186-5 18684671 10.1016/S1473-3099(08)70186-5 PMC7128517

[5] Ishola DA, Phin N. Could influenza transmission be reduced by restricting mass gatherings? Towards an evidence-based policy framework. J Epidmiol Globl Health. 2011;1:30–60. 10.1016/j.jegh.2011.06.004 PMC7104184 23856374

[6] Khan K, McNabb SJ, Memish ZA, Eckhardt R, Hu W, Kossowsky D, et al Infectious disease surveillance and modelling across geographic frontiers and scientific specialties. Lancet Infect Dis. 2012;12:222–230. doi: 10.1016/S1473-3099(11)70313-9 22252149 10.1016/S1473-3099(11)70313-9

[7] Shi P, Keskinocak P, Swann JL, Lee BY. The impact of mass gatherings and holiday traveling on the course of an influenza pandemic: a computational model. BMC Public Health. 2010;10:778 doi: 10.1186/1471-2458-10-778 21176155 10.1186/1471-2458-10-778 PMC3022852

[8] Weber TP, Stililanakis NI. Inactivation of influenza A viruses in the environment and modes of transmission: a critical review. J Infect. 2008;57:361–373. doi: 10.1016/j.jinf.2008.08.013 18848358 10.1016/j.jinf.2008.08.013 PMC7112701

[9] American Camp Association [Internet]. 2017 ACA Sites, Facilities, Programs Report. https://www.acacamps.org/press-room/aca-facts-trends.

[10] PricewaterhouseCoopers [Internet]. (January 2014). The Economic Significance of Meetings to the U.S. Economy; Interim Study Update for 2012. http://www.eventscouncil.org/Files/2012%20ESS/CIC%20Meetings%20ESS%20Update%20EXECUTIVE%20SUMMARY-FINAL.pdf.

[11] Qualls N, Levitt A, Kanade N, Wright-Jegede N, Dopson S, Biggerstaff M, et al Community Mitigation Guidelines to Prevent Pandemic Influenza—United States, 2017. MMWR Recomm Rep. 2017;66(No. RR-1):1–34. http://dx.doi.org/10.15585/mmwr.rr6601a1 10.15585/mmwr.rr6601a1 PMC5837128 28426646

[12] Rainey JJ, Phelps T, Shi J. Mass Gatherings and Respiratory Disease Outbreaks in the United States—Should We Be Worried? Results from a Systematic Literature Review and Analysis of the National Outbreak Reporting System. PLoS One [Internet]. 2016;11(8):e0160378 Available from: http://journals.plos.org/plosone/article?id=10.1371/journal.pone.0160378 10.1371/journal.pone.0160378 PMC4990208 27536770

[13] Jhung MA, Epperson S, Biggerstaff M, Allen D, Balish A, Barnes N, et al. Outbreak of Variant Influenza A(H3N2) Virus in the United States. Clin Infect Dis. 2013:57(12):1703–1712. doi: 10.1093/cid/cit649 24065322 10.1093/cid/cit649 PMC5733625

[14] Robinson S, Averhoff F, Kiel J, Blaisdell L, Haber M, Sites A, et al Pandemic influenza A in residential summer camps—Maine, 2009. Pediatr Infect Dis. 2012;31:547–550. 10.1097/INF.0b013e31824f8124 22414902

[15] Tsalik EL, Cunningham CK, Cunningham HM, Lopez-Marti MG, Sangvai DG, Purdy WK, et al. An Infection Control Program for a 2009 Influenza A H1N1 Outbreak in a University-based Summer Camp. J Am Coll Health. 2011;59(5): 419–426. doi: 10.1080/07448481.2010.534215 21500062 10.1080/07448481.2010.534215

[16] Blyth CC, Foo H, vanHal SJ, Hurt AC, Barr IG, McPhie K, et al Influenza outbreaks during World Youth Day 2008 mass gathering. Emerg Infect Dis. 2010;16(5): 809–815. doi: 10.3201/eid1605.091136 20409371 10.3201/eid1605.091136 PMC2953988

[17] Centers for Disease Control and Prevention (CDC). CDC Health Update: School (K-12) dismissal and childcare facilities: interim CDC guidance in response to human infections with the influenza A H1N1 virus, 2009. https://www.cdc.gov/h1n1flu/HAN/050109.htm.

[18] Glatman-Freedman A, Portelli I, Jacobs SK, Mathew JI, Slutzman JE, Goldfrank LR, et al Attack rates assessment of the 2009 pandemic H1N1 influenza A in children and their contacts: a systematic review and meta-analysis. PLoS One [Internet]. 2012; 7(11):e508228 Available from: http://journals.plos.org/plosone/article?id=10.1371/journal.pone.0050228 10.1371/journal.pone.0050228 PMC3523802 23284603

[19] World Health Organization (WHO). WHO recommendations for the post-pandemic period: pandemic (H1N1) 2009 briefing note 23, 2010. http://www.who.int/csr/disease/swineflu/notes/briefing_20100810/en/.

[20] Centers for Disease Control and Prevention (CDC).Update: influenza activity—United States, 2010–2011 season, and composition of the 2011–2012 influenza vaccine. MMWR. 2011;60(21):705–712. 21637185

[21] Saenz AC, Assaad FA, Cockburn WC. Outbreak of A2-Hong Kong-68 influenza at an international medical conference. Lancet. 1969;1(7585):91–93. 4178014 10.1016/s0140-6736(69)91104-0

[22] Botelho-Nevers E, Gautret P, Benarous L, Charrel R, Felkai P, Parola P. Travel-related Influenza A/H1N1 infection at a rock festival in Hungary: One virus may hide another one. J Travel Med. 2010;17:197–198. doi: 10.1111/j.1708-8305.2010.00410.x 20536890 10.1111/j.1708-8305.2010.00410.x

[23] Gutierrez I, Litsroth A, Hammadi S, Van Oyen H, Gerard C, Robesyn E, et al Community transmission of influenza A (H1N1)v virus at a rock festival in Belgium, 2–5 July. Euro Surveill. 2009;14(32). 10.2807/ese.14.31.19294-en 19660245

[24] Zieliński A. Evidence for excessive incidence of infectious diseases at mass gatherings with special reference to sporting events. Przegl Epidemiol. 2009;63:343–351. 19899589

[25] Gundlapalli AV, Rubin MA, Samore MH, Lopansri B, Lahey T, McGuire HL et al Influenza, Winter Olympiad, 2002. Emerg Infect Dis. 2006;12(1):144–146. doi: 10.3201/eid1201.050645 16494733 10.3201/eid1201.050645 PMC3291389

[26] Centers for Disease Control and Prevention (CDC). Surveillance for early detection of disease outbreaks at an outdoor mass gathering—Virginia, 2005. MMWR. 2006;55:71–74. 16437057

[27] Selig B, Hastings M, Cannon C, Allin D, Klaus S, Diaz FJ, et al Effect of Weather on Medical Patient Volume at Kansas Speedway Mass Gatherings. J Emerg Nurs. 2013;39(4):e39–44. doi: 10.1016/j.jen.2011.10.002 22204886 10.1016/j.jen.2011.10.002

[28] Centers for Disease Control and Prevention (CDC). Flu activity during the 2012–2013 Season. https://www.cdc.gov/flu/pastseasons/1213season.htm.

[29] Centers for Disease Control and Prevention (CDC). The flu season. https://www.cdc.gov/flu/about/season/flu-season.htm.

[30] Aiello AE, Coulborn RM, Perez V, Larson EL. Effect of hand hygiene on infectious disease risk in the community setting: a meta-analysis. Am J Public Health. 2008;98:1372–1381. doi: 10.2105/AJPH.2007.124610 18556606 10.2105/AJPH.2007.124610 PMC2446461

[31] Jefferson T, Del Mar CB, Dooley L, Ferroni E, Al-Ansary LA, Bawazeer GA, et al Physical interventions to interrupt or reduce the spread of respiratory viruses. Cochrane Database Syst Rev. 2011: CD006207 doi: 10.1002/14651858.CD006207.pub4 21735402 10.1002/14651858.CD006207.pub4 PMC6993921

[32] Vaughan E and Tinker T. Effective health risk communication about pandemic influenza for vulnerable populations. Am J Public Health. 2009;99 Suppl 2S324–S332. 10.2105/AJPH.2009.162537 PMC4504362 19797744

[33] Michael JA and Barbera JA. Mass gathering medical care: a twenty-five year review. Prehosp Disaster Med. 1997;12(4):305–12. 10179212

[34] Centers for Disease Control and Prevention (CDC). Flu prevention at a mass gathering. https://www.cdc.gov/nonpharmaceutical-interventions/gathering/index.html.

