# Supplementary material for: Estimating the frequency and characteristics of respiratory disease outbreaks at mass gatherings in the United States: Findings from a state and local health department assessment
Source: PLoS One. 2017 Oct 27;12(10):e0186730. doi: 10.1371/journal.pone.0186730 (PMC5659613; doi:10.1371/journal.pone.0186730)
Supplement: S1 Supplement — Copy of questionnaire used in online assessment. (DOCX) [file pone.0186730.s001.docx]

| **Form Approved**  **OMB Control No.: 0920-0879**  **Expiration Date: March 31, 2018**  **Mass Gathering Outbreak Information Collection Form**  **In collaboration with the Council of State and Territorial Epidemiologists (CSTE) and the National Association of County and City Health Officials (NACCHO), the U.S. Centers for Disease Control and Prevention (CDC) is conducting an assessment of mass gathering-related respiratory disease outbreaks reported in your jurisdiction from January 1, 2009 through December 31, 2014. For the purpose of this assessment, a mass gathering is considered a congregation (planned or unplanned) of 1000 or more persons in either an indoor or outdoor venue for a common purpose (e.g., sporting event, conference, state fair). An outbreak is one or more cases of a respiratory disease associated with a mass gathering (transmission linked to a point source, animal, or another person at the mass gathering). We are only interested in receiving aggregate, de-identified information about outbreaks and the mass gatherings with which they were associated that were not previously reported to CDC’s Notification of Outbreak Reporting System (NORS). The online assessment tool has two parts: 1) information on mass gathering-related respiratory disease outbreaks reported in your jurisdiction, and 2) information on relevant intervention and communication strategies implemented at mass gatherings. You will be able to report up to 10 mass gathering-related outbreaks. The assessment can be completed in about 30 minutes or less. Neither your name nor the name of your jurisdiction will be included in any report resulting from this assessment. CDC will collect and analyze all submitted data.**  **CDC estimates the average public reporting burden for this collection of information as 30 minutes per response, including the time for reviewing instructions, searching existing data/information sources, gathering and maintaining the data/information needed, and completing and reviewing the collection of information. An agency may not conduct or sponsor, and a person is not required to respond to a collection of information unless it displays a currently valid OMB control number. Send comments regarding this burden estimate or any other aspect of this collection of information, including suggestions for reducing burden to CDC/ATSDR Information Collection Review Office, 1600 Clifton Road NE, MS D-74, Atlanta, Georgia 30333; ATTN: PRA (0920-0879).**  **Assessment Instructions**  **To move from one page to the next, please use only the "Next" and "Previous" buttons found at the bottom of each screen. DO NOT use your browser's back and next button. If you accidentally click your browser's navigation button, you may be able to continue your assessment by pressing the F5 key or by refreshing the web page. Should you need to exit this assessment tool before completing it, you may save your existing answers by clicking on the "Stop" button below. When you are ready to resume, please click the link you received in the assessment invitation email. You will be able to continue where you left off answering.**  **Please provide the following contact information. This is used for recording purposes only.**  *Q1*  **Please check the jurisdiction you are currently representing:**  🔾 State  🔾 City or County  *Q2*  **State (if selected):**  ❑ Alabama  ❑ Alaska  ❑ Arizona  ❑ Arkansas  ❑ California  ❑ Colorado  ❑ Connecticut  ❑ Delaware  ❑ District of Columbia  ❑ Florida  ❑ Georgia  ❑ Hawaii  ❑ Idaho  ❑ Illinois  ❑ Indiana  ❑ Iowa  ❑ Kansas  ❑ Kentucky  ❑ Louisiana  ❑ Maine  ❑ Maryland  ❑ Massachusetts  ❑ Michigan  ❑ Minnesota  ❑ Mississippi  ❑ Missouri  ❑ Montana  ❑ Nebraska  ❑ Nevada  ❑ New Hampshire  ❑ New Jersey  ❑ New Mexico  ❑ New York  ❑ North Carolina  ❑ North Dakota  ❑ Ohio  ❑ Oklahoma  ❑ Oregon  ❑ Pennsylvania  ❑ Rhode Island  ❑ South Carolina  ❑ South Dakota  ❑ Tennessee  ❑ Texas  ❑ Utah  ❑ Vermont  ❑ Virginia  ❑ Washington  ❑ West Virginia  ❑ Wisconsin  ❑ Wyoming  *Q3*  **City or County (if selected):**  ❑ San Francisco Department of Public Health  ❑ Pinellas County Health Department  ❑ Louisville Metro Department of Public Health and Wellness  ❑ Boston Public Health Commission  ❑ Baltimore City Health Department  ❑ Columbus City Health Department  ❑ Montgomery County Department of Health  ❑ Metro Public Health Department  ❑ Memphis and Shelby County Health Department  ❑ Tacoma-Pierce County Health Department  ❑ Escambia County Health Department  ❑ Pasco County Health Department  ❑ Lincoln Trail District Health Department  ❑ Washoe County District Health Department  ❑ Knox County Health Department  ❑ Spokane Regional Health District  ❑ Norfolk City Health District  ❑ Maricopa County Department of Public Health  ❑ Alameda County Public Health Department  ❑ County of San Diego HHS and Public Health Services  ❑ Los Angeles County Department of Health Services - Public Health  ❑ Santa Clara County Public Health Department  ❑ Tri-County Health Department  ❑ Miami-Dade County Health Department  ❑ Hillsborough County Health Department  ❑ District 3, Unit 4: East Metro Health District  ❑ Chicago Department of Public Health  ❑ Southern Nevada Health District  ❑ New York City Department of Health and Mental Hygiene  ❑ Allegheny County Health Department  ❑ Philadelphia Department of Public Health  ❑ Houston Department of Health and Human Services  ❑ San Antonio Metropolitan Health District  ❑ Tarrant County Public Health Department  ❑ Public Health - Seattle and King County  *Q4*  **Position title:**  *Q5*  **Time with jurisdiction [years (ex. 5.3)]:**  .  **Please provide the following information about each mass gathering-related respiratory disease outbreak reported in your jurisdiction between January 1, 2009 and December 31, 2014 (not previously reported to NORS).**  *QMGath*  **Were there any mass gathering-related respiratory disease outbreaks in your jurisdiction between January 1, 2009 and December 31, 2014?**  🔾 Yes  🔾 No  *Q4*  **What was the name of the {first,second,third,etc...} mass gathering (if not known, please briefly describe):**  *Q5*  **{First,Second,Third,etc...} mass gathering location:   State:**  ❑ Alabama  ❑ Alaska  ❑ Arizona  ❑ Arkansas  ❑ California  ❑ Colorado  ❑ Connecticut  ❑ Delaware  ❑ District of Columbia  ❑ Florida  ❑ Georgia  ❑ Hawaii  ❑ Idaho  ❑ Illinois  ❑ Indiana  ❑ Iowa  ❑ Kansas  ❑ Kentucky  ❑ Louisiana  ❑ Maine  ❑ Maryland  ❑ Massachusetts  ❑ Michigan  ❑ Minnesota  ❑ Mississippi  ❑ Missouri  ❑ Montana  ❑ Nebraska  ❑ Nevada  ❑ New Hampshire  ❑ New Jersey  ❑ New Mexico  ❑ New York  ❑ North Carolina  ❑ North Dakota  ❑ Ohio  ❑ Oklahoma  ❑ Oregon  ❑ Pennsylvania  ❑ Rhode Island  ❑ South Carolina  ❑ South Dakota  ❑ Tennessee  ❑ Texas  ❑ Utah  ❑ Vermont  ❑ Virginia  ❑ Washington  ❑ West Virginia  ❑ Wisconsin  ❑ Wyoming  *Q5a*  **City:**  *Q6*  **Is this {first,second,third,etc...} mass gathering an annual event?**  🔾 Yes  🔾 No  🔾 Unknown  *Q7*  **{First,Second,Third,etc...} mass gathering dates month / year (enter starting month if covering more than one month): *(Enter MM/YYYY. Please estimate month and year if exact dates are not known)***  *Q7a*  **Estimated duration of mass gathering (in days):**  .  *Q8*  **For the {first,second,third,etc...} mass gathering reported, please provide the following information, if available.   {First,Second,Third,etc...} mass gathering type:**  🔾 Sporting event  🔾 Religious event  🔾 Professional conference  🔾 Trade show  🔾 Music event / concert  🔾 Fair (county or state)  🔾 Festival (e.g., cultural / neighborhood)  🔾 Camp  🔾 Political event (e.g., DNC / RNC)  🔾 Other (please describe): :  🔾 Unknown  *Q8a*  **Size of {first,second,third,etc...} mass gathering: *(Numeric estimate - e.g., 5000)***  *Q8b*  **Venue of {first,second,third,etc...} mass gathering:**  🔾 Indoor  🔾 Outdoor  🔾 Both  🔾 Unknown  *Q9*  **In the next two screens, please provide information on the type and size of the outbreak that occurred at the {first,second,third,etc...} mass gathering.   Approximate date of index case (first case identified) at {first,second,third,etc...} mass gathering: *(Enter MM/YYYY. Please estimate month and year if exact dates are not known)***  *Q10*  **Mode of transmission for {first,second,third,etc...} respiratory outbreak:**  🔾 Person to person (e.g., SARS)  🔾 Animal-borne (e.g., H3N2v)  🔾 Point source (e.g., Legionella)  🔾 Unknown  *Q12*  **Etiology: list agent(s) of {first,second,third,etc...} outbreak illnesses if known:**  ❑ Viral  ❑ Bacterial  ❑ Other :  🔾 Unknown  *Q12a*  **Name of agent(s) if known:**  *Q11b*  **Total number of total cases (probable and confirmed):**  *Q11c*  **Number of confirmed cases:**  *Q13*  **Age of cases of {first,second,third,etc...} outbreak: *(Check all that apply)***  ❑ Less than 2 years  ❑ 2 - 17 years  ❑ 18 - 64 years  ❑ 65 years or greater  🔾 Unknown  *Q14*  **Geographic spread of {first,second,third,etc...} outbreak: *(Check all that apply)***  ❑ Local  ❑ State-wide  ❑ National  🔾 Unknown  *Q999*  **Do you have any additional mass gathering-related outbreaks to report in this assessment?**  🔾 Yes  🔾 No  **Please briefly describe the last time since January 1, 2009 your jurisdiction implemented (supported with human or financial resources) the following during a mass gathering.**  *Q15d*  **Conducted on-site infectious disease surveillance (data collection/monitoring of disease/syndromic events)?**  🔾 Yes  🔾 No  🔾 Don't know / unsure  *Q15b*  **Type of Mass Gathering (e.g., sporting event):**  ❑ Sporting event  ❑ Religious event  ❑ Professional conference  ❑ Trade show  ❑ Music event / concert  ❑ Fair (county or state)  ❑ Festival (e.g., cultural / neighborhood)  ❑ Camp  ❑ Political event (e.g., DNC / RNC)  ❑ Other  ❑ Unknown  *Q15a*  **When (Enter month / year – MM/YYYY. Please estimate month and year if exact dates are not known):**  *Q15c*  **Why (please provide a brief description):**  *Q16d*  **Implemented any nonpharmaceutical interventions (e.g., hand washing stations, provision of tissues for appropriate cough etiquette, social distancing)?**  🔾 Yes  🔾 No  🔾 Don't know / unsure  *Q16b*  **Type of Mass Gathering (e.g., sporting event):**  ❑ Sporting event  ❑ Religious event  ❑ Professional conference  ❑ Trade show  ❑ Music event / concert  ❑ Fair (county or state)  ❑ Festival (e.g., cultural / neighborhood)  ❑ Camp  ❑ Political event (e.g., DNC / RNC)  ❑ Other  ❑ Unknown  *Q16a*  **When (Enter month / year – MM/YYYY. Please estimate month and year if exact dates are not known):**  *Q16aa*  **Which type of nonpharmaceutical interventions: *(Check all that apply)***  ❑ Increased number of hand washing stations  ❑ Distribution of hand sanitizer  ❑ Distribution of tissues  ❑ Increased of spacing of attendees (e.g., seating arrangements) social distancing  *Q16c*  **Why (please provide a brief description):**  *Q17d*  **Conducted any public health messaging (e.g., radio / TV announcements, posters, twitter feeds)?**  🔾 Yes  🔾 No  🔾 Don't know / unsure  *Q17b*  **Type of Mass Gathering (e.g., sporting event):**  ❑ Sporting event  ❑ Religious event  ❑ Professional conference  ❑ Trade show  ❑ Music event / concert  ❑ Fair (county or state)  ❑ Festival (e.g., cultural / neighborhood)  ❑ Camp  ❑ Political event (e.g., DNC / RNC)  ❑ Other  ❑ Unknown  *Q17a*  **When (Enter month / year – MM/YYYY. Please estimate month and year if exact dates are not known):**  *Q17aa*  **Which type of messaging: Please indicate media used and/or brief description of content *(Check all that apply)***  ❑ Posters  ❑ Fact sheets  ❑ Guidance  ❑ Planning checklists  ❑ Traditional media (TV / Radio)  ❑ Web links (or widgets)  ❑ Social media messages  ❑ Mobile phone text messages and apps  ❑ Other (please describe): :  *Q17c*  **Why (please provide a brief description):**  *Q18d*  **Implemented any other public health activities before or during a mass gathering in order to prevent or slow transmission of respiratory infections (e.g., an influenza vaccination clinic)?**  🔾 Yes  🔾 No  🔾 Don't know / unsure  *Q18b*  **Type of Mass Gathering (e.g., sporting event):**  ❑ Sporting event  ❑ Religious event  ❑ Professional conference  ❑ Trade show  ❑ Music event / concert  ❑ Fair (county or state)  ❑ Festival (e.g., cultural / neighborhood)  ❑ Camp  ❑ Political event (e.g., DNC / RNC)  ❑ Other  ❑ Unknown  *Q18a*  **When (Enter month / year – MM/YYYY. Please estimate month and year if exact dates are not known):**  *Q18aa*  **Describe other activity that has been implemented:**  *Q18c*  **Why (please provide a brief description):**  *Q21*  **What can CDC do to help improve our communication with you about mass gatherings, pandemic preparedness and nonpharmaceutical interventions? *(Please provide any comments or suggestions)*** |
| --- |
